# Shotgun Metagenomics Reveals Microbial Diversity, Resistome, and Plasmidome in Dairy Cattle Feces

**DOI:** 10.3390/vetsci13030275

**Published:** 2026-03-16

**Authors:** Shehla Shehla, Muhammad Kashif Obaid, Sadaf Niaz, Munir Ahmad Khan, Anum Ali Ahmad, Mostafa A. Abdel-Maksoud, Abdulaziz Alamri, Salman Alrokayan, Muhammad Shoaib, Sumaira Shams, Qiaoyun Ren

**Affiliations:** 1 Department of Zoology, Abdul Wali Khan University, Garden Campus Mardan, Mardan 23200, Pakistan; shehla@awkum.edu.pk (S.S.); sadaf@awkum.edu.pk (S.N.); 2 State Key Laboratory of Animal Disease Control and Prevention, Lanzhou Veterinary Research Institute, College of Veterinary Medicine, Lanzhou University, Chinese Academy of Agricultural Sciences, Lanzhou 730046, China; kashifobaid@awkum.edu.pk; 3 Key Laboratory of Veterinary Parasitology of Gansu Province, Gansu Province Research Center for Basic Disciplines of Pathogen Biology, Lanzhou 730046, China; 4 Department of Medicine, Gomal Medical College, Dera Ismail Khan 29050, Pakistan; munirwensam@gmail.com; 5 The Roslin Institute, The University of Edinburgh, Easter Bush Campus, Edinburgh EH25 9RG, UK; aahmad3@ed.ac.uk; 6 Research Chair of Biomedical Applications of Nanomaterials, Biochemistry Department, College of Science, King Saud University, P.O. Box 2455, Riyadh 11451, Saudi Arabia; mabdmaksoud@ksu.edu.sa (M.A.A.-M.); salrokayan@ksu.edu.sa (S.A.); 7 Biochemistry Department, College of Science, King Saud University, P.O. Box 2455, Riyadh 11451, Saudi Arabia; abalamri@ksu.edu.sa; 8 Jiangsu Co-Innovation Center for Prevention and Control of Important Animal Infectious Diseases and Zoonoses, College of Veterinary Medicine, Yangzhou University, Yangzhou 225009, China; 9 Hebei Key Laboratory of Animal Physiology, Biochemistry and Molecular Biology, Hebei Collaborative Innovation Center for Eco-Environment, Ministry of Education, Key Laboratory of Molecular and Cellular Biology, College of Life Sciences, Hebei Normal University, Shijiazhuang 050024, China

**Keywords:** cattle feces, metagenomics, microbial diversity, resistome, plasmidome, Pakistan

## Abstract

Fecal microbiota are shaped by upstream digestive processes and reflect the outcome of host–microbe interactions, including the resistant microbial fraction that survives to be excreted. This is particularly crucial for assessing zoonotic risks and environmental contamination, as feces are the primary source of dissemination, which is considered an emerging One Health threat. Therefore, we conducted a pilot study to obtain the exploratory findings regarding the cattle GIT microbial composition, potential resistome, and their transmission drivers, such as plasmids, using metagenomic analysis from different districts in Khyber Pakhtunkhwa (KP) province, Pakistan. For this purpose, a total of 150 fecal samples (50 from each district) of healthy cattle were collected from various farms in Mardan (FC1), Peshawar (FC2), and Dera Ismail Khan (FC3) districts. Total DNA from each sample was extracted, pooled (FC1, FC2, and FC3), and sequenced via the Illumina platform. Bacteria were the highly abundant kingdom, while Pseudomonadota and Bacillota were dominant phyla in all samples. *Caryophanon latum* and *Escherichia coli* were highly abundant at the species level. A large resistome (40–49 genes), including critical genes, such as *tet*(X), *bla*_OXA-427_, and plasmidomes (16–22), such as IncF, was detected in the samples. The prominence of certain commensal or opportunistic pathogens in the fecal microbiota may indicate the presence of sub-clinical gastrointestinal disruptions or disease that may affect cattle herds. The fecal resistome is extensive, identifying dairy cattle in these regions as important reservoirs for AMR genes capable of spreading via HGT. This pilot study establishes that the fecal microbiota of dairy cattle in this region are not merely a waste product but a complex ecosystem, rich in microbiota of One Health significance.

## Simple Summary

Gastrointestinal tract (GIT) microbiota play a crucial role in maintaining health and improving the production performance of livestock. While fecal samples do not directly represent these foregut communities, they provide a highly relevant and practical snapshot of the terminal gastrointestinal ecosystem. Upstream digestive processes shape the fecal microbiota and reflect the outcome of host–microbe interactions, including the resistant microbial fraction that survives to be excreted. This is particularly crucial for assessing zoonotic risks and environmental contamination, as feces are the primary source of dissemination. However, metagenomic data on livestock fecal microbiota in Pakistan remain scarce. This study explored the microbial composition, resistome, and plasmidome in cattle feces from three districts in Khyber Pakhtunkhwa, Pakistan, via a metagenomics approach. Bacteria were highly abundant (84.00–91.00%) at the kingdom level, followed by viruses (2.00–4.00%), archaea (0.20–1.00%), and Eukaryota (0.02–0.06%). *Escherichia coli*, *Providencia stuartii*, and *Aliarcobacter skirrowii* were highly abundant at the species level in feces, suggesting a GIT environment that may be conducive to the colonization of commensal and opportunistic pathogens of human and animal health concern. We observed that the highest number of species was in Peshawar (FC2; *n* = 9147), followed by Mardan (FC1; *n* = 8481) and Dera Ismail Khan (FC3; *n* = 8462), which may reflect underlying variations in farm management practices, local antibiotic usage, dietary compositions, or environmental exposures. A large resistome (40–49 genes) and plasmidome (16–22) were detected from all regions, indicating an active hotspot for horizontal gene transfer. In conclusion, this pilot study establishes that the fecal microbiota of dairy cattle in this region are not merely a waste product but a complex ecosystem rich in microbiota of One Health significance.

## 1. Introduction

Ruminants are economically important livestock because of their unique ability to convert human-indigestible plant biomass into food products for human beings [1,2,3]. A large number of microbial populations exist in the gastrointestinal tract (GIT) of animals [4]. These microbes are involved in the maintenance of animal health, and slight changes in their composition can negatively impact the animal’s health and well-being, resulting in disturbance of their overall productivity [5,6,7]. Specifically, intestinal microbiota have numerous beneficial health effects, like providing beneficial nutrients, converting metabolites, and having mutualistic interactions with host cells [6,8,9,10]. Understanding the complex interactions and composition of these communities is crucial for developing strategies to optimize feed efficiency and mitigate disease-causing agents to improve the health of livestock [11]. Various factors, including geography, diet, and management practices, can significantly influence the composition and function of the GIT microbiota in cattle [12]. Carbohydrate-Active Enzymes (CAZymes) play a crucial role in the digestion of complex carbohydrates in cattle, and their presence in fecal samples can provide insights into the microbial community’s fibrolytic capabilities [13,14]. CAZymes in fecal samples are important for understanding host genetics and diet effects on enzyme diversity, exploring novel enzymes, and identifying potential probiotic strains [13,15,16].

Disturbances in the GIT microbiota can lead to severe digestive and metabolic disorders in cattle [17]. Additionally, pathogenic bacteria in feces can pose risks for human foodborne illnesses, as well as cause environmental contamination [18,19]. Livestock manure from cattle and poultry is a significant reservoir of pathogenic microorganisms and parasite eggs, posing a substantial risk for zoonotic disease transmission [19,20]. Feces can harbor dangerous pathogens, such as *Salmonella*, *Bacillus anthracis*, and *Clostridium botulinum*, which threaten human health through direct contact, aerosolized droplets, or contamination of food and water supplies [21,22,23,24]. Among these pathogens, *Escherichia* (*E.*) *coli* is an important reservoir of resistance genes and plasmids, which play a crucial role in the dissemination of such genes through horizontal gene transfer [25].

Relying on culturing methods provides a foundational but fundamentally incomplete assessment of microbial communities associated with livestock [26,27,28]. Additionally, culturing methods are insufficient for getting the GIT “dark matter” (microbes that are unable to grow on the culture plates in the lab). The advent of next-generation sequencing (NGS) addresses the limitations of traditional culturing methods by enabling direct sample analysis through metagenomics [29]. This approach offers comprehensive insights into the microbial composition and their interactions with host animals and the surrounding environment [29]. NGS-based studies clarify the identification of both known and previously uncharacterized microbial species, as well as their functional annotations. Most of the studies focus on 16S rRNA sequencing, which provides only taxonomic information regarding the bacterial and archaeal communities without any direct functional analysis. Shotgun metagenomics overcomes many limitations of targeted approaches by sequencing all the DNA present in a sample rather than amplifying any specific gene regions. This comprehensive approach allows the detection of a broader range of microorganisms along with their functional profiles, providing a direct link between community structure and functions [30]. Minor alterations in microbial diversity, when found in very small quantities, can be evaluated via shotgun metagenomics studies [31]. Metagenomic analysis of fecal samples from dairy cattle has been previously documented [32,33,34]. However, data regarding its associated microbial diversity in Pakistan remains limited. Furthermore, the NGS-based studies for antibiotic-resistant gene identification in cattle’s fecal samples have been performed in Indonesia and China [35,36].

Pakistan is an agricultural country, and livestock production plays a significant role in the country’s economy. Cattle are vital for subsistence, dairy, and draught power, which are typically raised under distinct regional climates and fed on local, roughage-based diets, such as crop residues. There is a critical knowledge gap regarding the composition of the fecal microbiota of cattle in Pakistan, particularly across different geographical regions. This is particularly crucial for assessing zoonotic risks and environmental contamination, as feces are the primary source of dissemination and are considered an emerging One Health threat. Therefore, this pilot study aimed to provide the first comprehensive microbial taxonomic inventory, resistome, and plasmidome in cattle feces from three districts in Khyber Pakhtunkhwa. The selected districts, Mardan, Peshawar, and Dera Ismail Khan, differ markedly in climate, vegetation, and prevailing farming practices, factors that are hypothesized to influence the fecal microbiota of cattle.

## 2. Materials and Methods

### 2.1. Ethical Approval

The proposed research study, including all its protocols and opted procedures, was approved by the graduate studies committee at the Department of Zoology, Abdul Wali Khan University, Mardan (No: Dir/A&R/AWKUM/2025/362, Approval Date: 21 January 2025). All the experimental work was performed according to the approved guidelines and regulations of the committee. Written and oral permission were obtained from the animal owner during the sampling.

### 2.2. Study Area and Sampling

**Figure 1:**
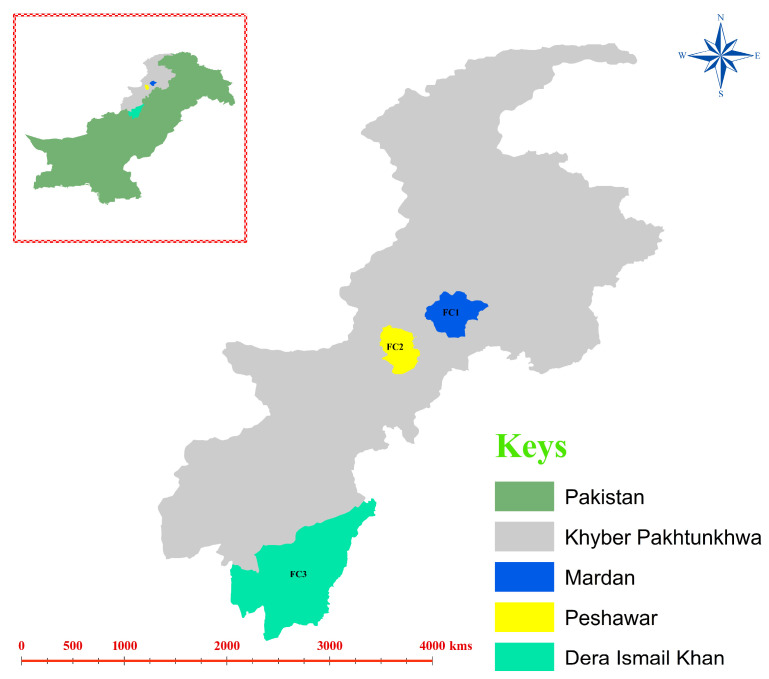
A study map presenting the sampling districts (FC1: Mardan, FC2: Peshawar, and FC3: Dera Ismail Khan). The map was created using ArcGIS 10.3.1 (https://desktop.arcgis.com/en/, accessed on 12 October 2025).

Three districts in KP province, including Mardan (34.189862, 72.045405), Peshawar (33.997110, 71.546226), and Dera Ismail Khan (31.858685, 70.886773), were selected for sampling (Figure 1). Three different cattle farms (a total of 9 farms) in each district were selected for fecal sample collection. The cattle breed (Holstein_Friesian) looked physically healthy as local veterinary officials were visiting these farms in respective districts to examine their health status, and no specific physical examination assessment was performed before sampling. Unhealthy cattle were excluded from sampling for this study. However, no information regarding the usage of antibiotic(s) or medication(s) was collected during sampling. According to the farm owners, the cattle were allowed to roam freely in search of pasture and food during the morning. At night, they were confined near the owners’ residences and fed a basic diet of grasses and other similar fodder. The sanitary conditions of farms were clean, dry, and consisted of well-drained bedding that was regularly cleaned to keep the farm environment hygienic. Excellent ventilation to minimize the respiratory pathogens and maintain fresh air inside the farm was also maintained. Feeding and watering systems were also kept free of molds, manure, and spoilage through daily cleaning of water tanks, while feed storage was protected from pests. Overall, 150 fecal samples (50 from each district, as 17 from farm-1, 17 from farm-2, and 16 from farm-3) were collected from different cattle in the targeted farms during the winter season, and sampling from each cattle was performed only once. Specifically, the central part of each fecal sample (~500 mg) of individual cattle was collected immediately after their first defecation during the morning (after visual monitoring of each cattle), to minimize its contact with soil or other secondary sources. A single-use wooden sterile spatula and single-use latex gloves were used to collect each fecal sample, which were discarded after the first use. This ensured that each fecal sample was collected with fresh, sterile instruments, making any cross-contamination between samples impossible. Individual stool samples were immediately placed in sterile bags (Nasco, Fort Atkinson, WI, USA) and shipped on ice to the laboratory of the Department of Zoology, within 24 h, and immediately stored at −80 °C till further processing.

### 2.3. DNA Processing, Library Construction, Quality Control, and Sequencing

Total genomic DNA (gDNA) was extracted from ~200 mg fecal samples individually using commercially available E.Z.N.A.^®^ Stool DNA Kit (Omega BIO-TEK, D4015-02, Norcross, GA, USA), according to the manufacturer’s instructions. The integrity of gDNA was detected by 1% agarose gel electrophoresis, and quantification was performed via Nanodrop 2000 spectrophotometer (Thermo Fisher Scientific, Waltham, MA, USA). Due to budgetary constraints, individual gDNA from 50 cattle from each district were equimolarly pooled into a single composite sample per district (FC1, FC2, and FC3 for Mardan, Peshawar, and Dera Ismail Khan, respectively) and preserved at −80 °C till further processing.

A total of 1 μg of genomic DNA (gDNA) was randomly fragmented into segments of about 350 base pairs by employing a Covaris ultrasonic disruptor (Covaris, LLC, Woburn, MA, USA), enabling library construction. The thorough library preparation via Rapid Plus DNA Lib Prep Kit for Illumina (RK20208, ABclonal, Wuhan, China) included various levels like end repair, A-tail addition, sequencing adaptor ligation, purification, and PCR amplification. Subsequently, the integrity of library fragments and the lengths of inserted fragments were evaluated using AATI analysis and Qubit 2.0 Fluorometer (ThermoFisher Scientific, Waltham, MA, USA), after which the library was diluted to 2 ng/µL. The Agilent 2100 (Agilent Technologies Inc., Santa Clara, CA, USA) was used to determine the insert size of the library. After establishing the required insert size, the precise concentration of the effective library was determined using qPCR (effective library concentration > 3 nM) to evaluate library quality. After successfully assessing the library’s quality, several libraries were aggregated based on their optimum concentrations and target data output specifications and then processed for sequencing on an Illumina PE150 sequencer (Novogene Co., Ltd., Beijing, China).

### 2.4. Data Pre-Processing and Assembly Preparation

Raw data reads were processed for adapter trimming and low-quality reads by using Fastp v.0.23.1 (https://github.com/OpenGene/fastp, accessed on 20 October 2025) to generate clean data for further analysis. When one or both of paired reads included adapter contamination, more than 10% of reads contained unknown nucleotides, or more than 50% of reads contained poor quality nucleotides (base quality less than 5), they were rejected and excluded from proceeding to further analysis. Clean data from each sample were blasted against the host database to exclude host reads, addressing potential host contamination in each sample. By default, the following parameter settings were used with the Bowtie2 software v.2.5.4 (http://bowtie-bio.sourceforge.net/bowtie2/index.shtml, accessed on 5 November 2025): end-to-end, sensitive, I 200, and X 400 [37,38,39]. Clean data were investigated using MEGAHIT software v.1.2.9, which had the subsequent assembly parameter settings: meta-large presets (sensitive, end-to-end, I 200, X 400) [37,40], and the scaffolds that originated from the N junction were broken to produce scaffolds lacking N [41,42].

### 2.5. Gene Prediction and Abundance Analysis

MetaGeneMark v.2.1 (http://topaz.gatech.edu/GeneMark/, accessed on 10 November 2025) was implemented to predict the ORF for scaftigs (≥500 bp) of each sample using the default configuration [38,43,44,45,46], and the predicted results were filtered to exclude data with a length of less than 100 nucleotides [40,42,47,48,49]. For ORF prediction, CD-HIT software v.4.5.8 (http://www.bioinformatics.org/cd-hit/, accessed on 15 November 2025) was used to eliminate redundancy [50,51] and acquire the non-redundant starting gene catalogue with different parameter settings: -c 0.95, -G 0, -aS 0.9, -g 1, and -d 0 [43,46,48]. Bowtie2 v. 2.5.4 was used to determine the number of gene reads for each sample alignment using various parameter settings (end-to-end, sensitive, I 200, X 400) after the clean data from each sample were aligned to the original gene catalogue [42,43]. To define the gene catalogue (unigenes) for further analysis, genes having reads ≥2 in each sample were filtered out [48]. The abundance of each gene in each sample was estimated using a formula according to previous studies [52,53,54]. Utilizing the abundance of each gene in the gene catalogue across samples, we conducted core–pan gene analysis via R Software v.2.15.3 (Package: ggplot2) and Venn diagram analysis via R software v.3.0.3 (Package: VennDiagram) for gene quantity.

### 2.6. Species Annotation

DIAMOND software v.2.1.9 (https://github.com/bbuchfink/diamond/, accessed on 23 November 2025) was applied to align unigenes sequences with the Micro_NR database v. 2.1.9, which comprises sequences from bacteria, fungi, archaea, and viruses retrieved from NCBI’s NR database (https://www.ncbi.nlm.nih.gov/, accessed on 27 November 2025). Alignment was executed with the blastp approach, with a parameter configuration of 1 × 10^−5^ [37,52]. From alignment outcomes for each sequence, the ones with an e-value ≤ the minimum e-value multiplied by 10 were chosen. Given that each sequence may yield different alignment results, the lowest common ancestor (LCA) technique was used inside the systematic taxonomy of MEGAN version 6.0 software [55]. A species’ abundance in each sample was determined by adding the abundance of all genes corresponding to that species [38,43,56]. To evaluate microbial richness across taxonomic hierarchies and sample groups, we performed alpha-diversity analysis using observed species counts and richness estimators—abundance-based coverage estimator (ACE) and Chao1. Additionally, to study the similarity among samples, a cluster tree of samples was constructed by performing cluster analysis on each sample using the Bray–Curtis distance, one of the most commonly used distance indicators. The gene count of a species in each sample was equivalent to the count of genes with non-zero abundance among those designated as that species.

### 2.7. Detection of Resistance Genes and Plasmids

The resistome and plasmidome in all samples were detected using the ResFinder and PlasmidFinder tools in the Abricate package (https://github.com/tseemann/abricate, accessed on 2 December 2025). The total number of resistance genes and plasmids identified in each sample was also determined.

### 2.8. Carbohydrate-Active Enzymes (CAZymes) Metabolic Pathways

The CAZy database, a professional-grade database for the study of carbohydrate-active enzymes, mainly covers 6 functional categories, including glycoside hydrolases (GHs), glycosyl transferases (GTs), polysaccharide lyases (PLs), carbohydrate esterases (CEs), Auxiliary Activities (AAs), and Carbohydrate-Binding Modules (CBMs). In the current study, functional annotation and abundance analysis of CAZy (http://www.cazy.org/, accessed on 12 December 2025) were followed [57] by aligning the unigenes in DIAMOND software v.2.1.9 (https://github.com/bbuchfink/diamond/, accessed on 18 December 2025) and selecting the filtered comparison result with the highest score (one HSP > 60 bits) for subsequent analysis [43,46,58,59]. From the functional annotation results and gene abundance table, the number of genes at each classification level for each sample was obtained, and the number of genes with a function in a particular sample was equal to the number of genes with an abundance of not 0 in the genes annotated as that function.

## 3. Results

### 3.1. Overview of Sequencing Data

In this study, 16,300 Mbp of raw data were generated using the Illumina sequencing platform. After quality control, 16,030 Mbp of clean data were obtained. The average effective generated data was 98.39%. At the same time, further results regarding quality score distribution along reads, as well as base content along reads for each respective pool sample, are presented in Table S1. The total length in bp of each sample, scaftigs number, average length in bp, N50 length in bp, N90 length in bp, and maximum length in bp are shown in Table 1. After obtaining the assembly results, the MetaGeneMark software was used for gene prediction, as mentioned in the methodology section. Further, basic gene catalogue information statistics were obtained, as shown in Table S2. A total of 797,541 ORFs were identified, and the number of complete genes in the overall ORFs was 226,442, accounting for 28.39% of the ORFs (Table S2).

### 3.2. Core–PAN Gene

Various numbers of genes were obtained in each respective pool sample, based on the existence of abundant genes, and presented in a petal map. The results showed 14,486, 32,234, and 130,331 genes were specifically available in FC1, FC2, and FC3 samples, respectively. In addition, 118,297 genes co-existed in FC1 and FC2, and 66,016 genes co-existed in FC2 and FC3, while 46,018 co-existed in FC1 and FC3 samples. Almost 248,137 genes were present in all the aforementioned three samples (Figure S1).

### 3.3. Fecal Microbial Composition

**Figure 2:**
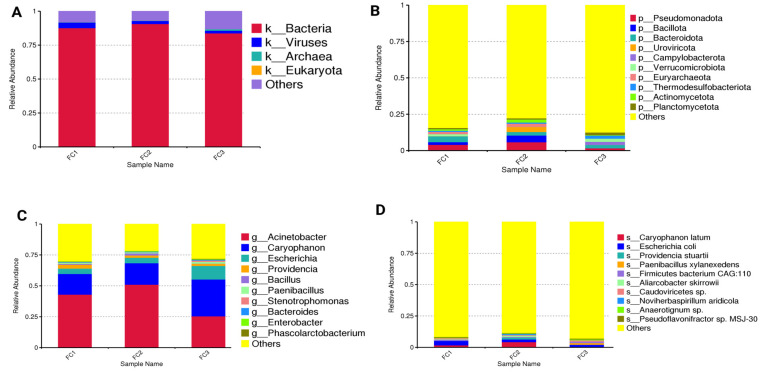
The bar chart shows the relative abundance of fecal microbiota at (**A**): kingdom, the others’ category represents sequences that could not be confidently classified at this level, (**B**): phylum, (**C**): genus, and (**D**): species levels. FC1: Mardan, FC2: Peshawar, and FC3: Dera Ismail Khan.

Fecal microbial composition at different taxonomic levels was evaluated (Figure 2A–D and Figures S2–S4). The top 10 taxa having the highest relative abundance in each sample (FC1, FC2, and FC3) were identified, while the remaining taxa were compiled as others. At the kingdom level, bacteria (84.00% to 91.00%) were dominant, followed by viruses (2.00–4.00%), archaea (0.20–1.00%), and Eukaryota (0.02–0.06%). At the phylum level, Pseudomonadota (25.00–51.00%), Bacillota (17.00–30.00%), and Bacteroidota (4.00–11.00%) were the dominant phyla. Other minor phyla included Uroviricota (2.00–4.00%), Campylobacterota (0.10–2.00%), and Verrucomicrobiota (0.80–1.00%). Phylum Pseudomonadota was highly abundant in FC1 (43.00%) and FC2 (51.00%), while Bacillota showed higher abundance in FC3 (30.00%). At the genus level, *Acinetobacter* (2.00–6.00%), *Caryophanon* (0.01–5.00%), and *Escherichia* (2.00–4.00%) were the dominant genera. Genus *Providencia* (0.02–3.00%), *Bacillus* (0.04–4.00%), and *Paenibacillus* (0.05–2.00%) were the minor genera in all samples. At the species level, *Caryophanon* (*C.*) *latum* (0.01–5.00%), *E. coli* (2.00–4.00%), Firmicutes bacterium CAG:110 (unclassified species in phylum Firmicutes; 1.00–2.00%), and *Providencia* (*P.*) *stuartii* (0.02–1.00%) were dominant in all samples. Genus *Acinetobacter* (6.00%) and species *C. latum* (5.00%) showed higher abundance in FC2.

### 3.4. Fecal Microbial Diversity

**Figure 3:**
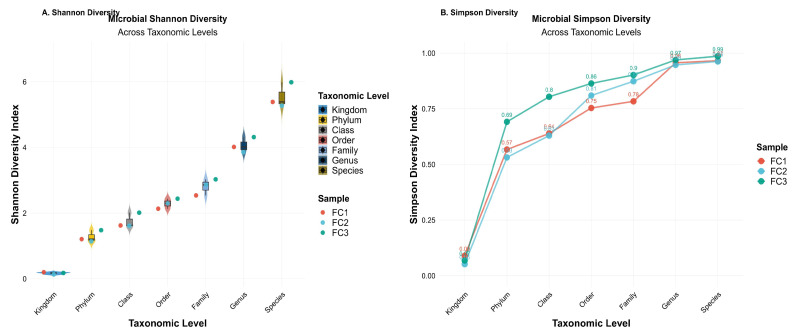
Microbial alpha diversity across each taxonomic level in three samples (FC1: Mardan, FC2: Peshawar, and FC3: Dera Ismail Khan). (**A**) Shannon diversity index. (**B**) Simpson diversity index.

We calculated Shannon and Simpson diversity indices at different taxonomic levels to measure the richness and evenness of microbial taxa in all samples (Figure 3). The Shannon diversity index showed a progressive increase in species richness and evenness from higher to lower taxonomic levels, with the highest diversity observed at the species level (Figure 3A). The Simpson diversity index gives more weight to the most abundant species, making it less sensitive to rare taxa in the samples. Values closer to 1 indicate high diversity. Simpson’s diversity index followed a similar enhancing trend, demonstrating greater evenness at finer taxonomic resolutions, as shown in Figure 3B. At the kingdom level, only four taxa were observed in all processed samples. However, the number of observed taxa increased markedly at finer taxonomic levels. A heatmap of observed microbial taxa across these three samples showed that FC2 had the highest number of observed species (*n* = 9147), followed closely by FC1 (*n* = 8481) and FC3 (*n* = 8462), as shown in Figure S5.

**Figure 4:**
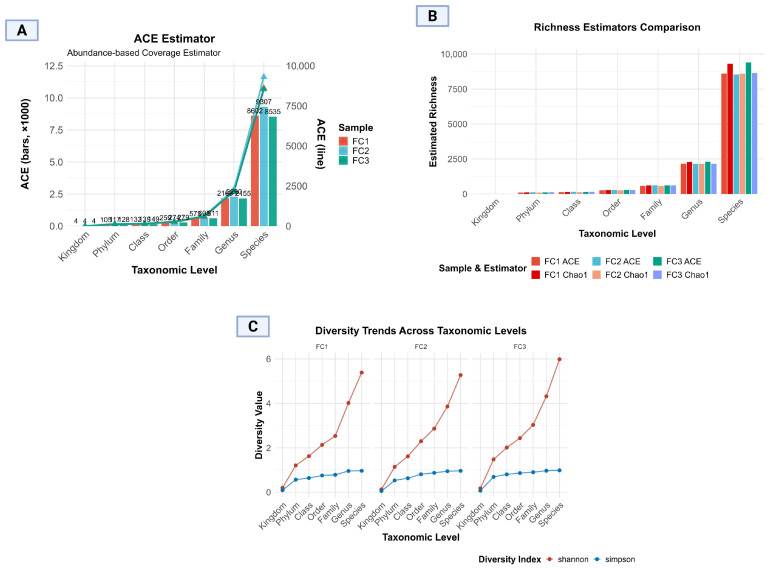
Microbial alpha diversity across taxonomic levels in three samples (FC1: Mardan, FC2: Peshawar, and FC3: Dera Ismail Khan). (**A**) Abundance-based coverage estimator, (**B**) richness estimator comparison, and (**C**) Shannon and Simpson diversity trends across taxonomic levels.

Further analysis presented various other aspects, as shown in Figure 4A, which demonstrated the ACE estimator regarding the progressive increase in microbial richness, with minimal richness at the kingdom level (*n* = 4) and maximal richness at the species level (FC1; 8602, FC2; 9307, FC3; 8535). The bar and line plot presentation clearly illustrates consistency in richness estimates within each sample group, while also highlighting minimal inter-sample variation at lower taxonomic levels, and it becomes more prominent at genus and species levels. Notably, these slight variations indicate measurable differences in microbial community richness across the samples, highlighting the differences in the GIT of cattle of targeted localities. These minor discrepancies reflect the differences in algorithm sensitivity: ACE tends to be more responsive to abundant species. The similarity in values, however, suggests a well-sampled and representative microbial data set (Figure 4A–C).

**Figure 5:**
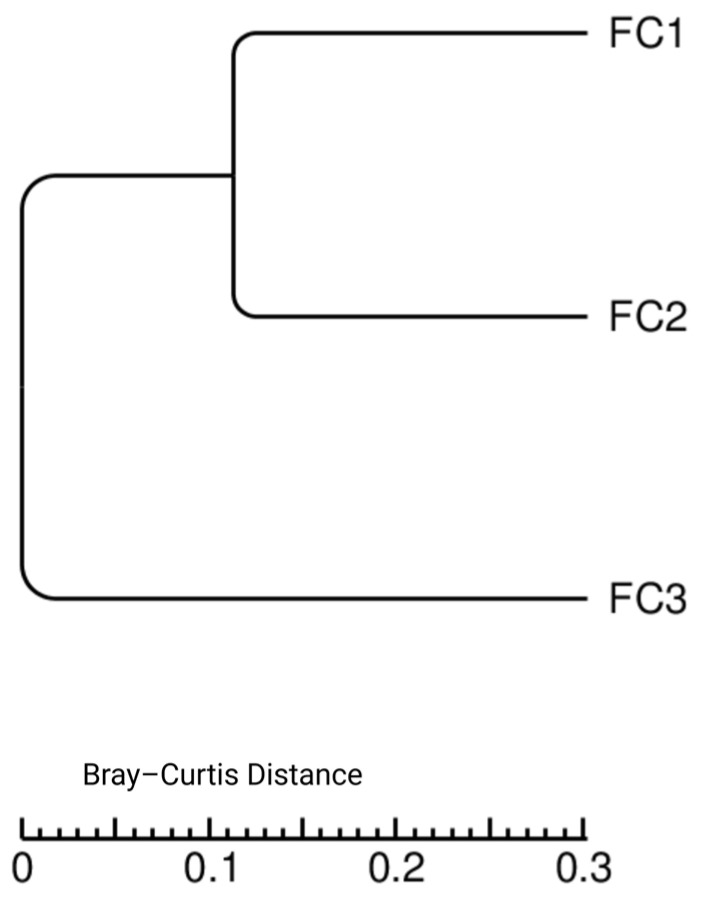
A dendrogram showing the Bray–Curtis distance clustering of the three samples. FC1: Mardan, FC2: Peshawar, and FC3: Dera Ismail Khan.

Bray–Curtis dissimilarity was used to explore compositional differences among individual samples from the three districts. As shown in Figure 5, the FC1 and FC2 samples showed greater similarity to each other compared to FC3, indicating variation in microbial composition among the three samples. However, given the pilot nature of this study and the absence of biological replication, we did not perform any statistical tests and used this image for exploratory purposes.

### 3.5. Antimicrobial Resistance Genes and Plasmids

**Figure 6:**
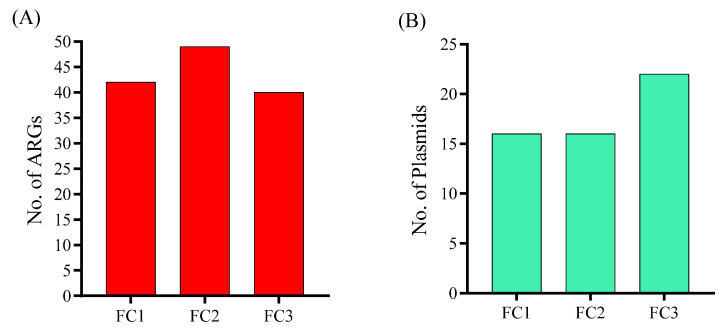
(**A**) Total number of antimicrobial resistance genes (ARGs) acquired by each sample. FC1: Mardan, FC2: Peshawar, and FC3: Dera Ismail Khan. (**B**) Total number of plasmids (ARGs) acquired by each sample.

Further investigation of the samples using Resfinder and Plasmidfinder in Abricate revealed that our samples carried a large number of resistance genes, with FC1 = 42, FC2 = 49, and FC3 = 40 genes (Figure 6A). Similarly, the plasmidome analysis revealed that the FC3 sample carried a higher number of plasmids (*n* = 22) compared to FC1 (*n* = 16) and FC2 (*n* = 16) (Figure 6B). The specific resistance genes analysis revealed that samples were carrying shared genes belong to different classes, such as *cfxA3*, *bla*_TEM-1B_, *bla*_CTX-M-15_, *bla*_OXA-235_, *bla*_OXA-396_, and *bla*_SHV-187_ in beta-lactam class, *aph(3″)-Ib* and *aph(6)-Id* in aminoglycoside, *sul1* and *sul2* in sulfonamide, *dfrA1* and *dfrA14* in trimethoprim, *oqxA*, *oqxB*, *qnrB4*, and *qnrS1* in fluoroquinolones, *catB1* and *catB7* in phenicol class, and other genes mentioned in Table 2. Most importantly, all of the samples were identified with the critical resistance gene *tet*(X) that confers resistance to tigecycline, a last-resort antibiotic. The identity, coverage, and contig-level context of each detected gene in all samples are listed in Table S3.

Further detailed plasmidome analysis confirmed that all samples predominantly carried Col and IncF family plasmids. However, plasmids belonging to other families, such as IncI, IncY, and IncR, were also detected. Among the Col family plasmids, Col440I, Col440II, ColRNAI, and ColpVC were detected in all samples. Similarly, IncFIA, IncFIA(HI1), IncFIB(AP001918), and IncFIB(K) types were detected in all samples, along with different replicon types of IncFII. The FC3 sample was carrying some additional plasmid types, such as Col156, Col(BS512), Col(IMGS31), Col(IRGK), and IncX1 (Table 2). Additionally, we also detected *qnrB19* in a Col440I plasmid-associated contig in the FC3 sample.

### 3.6. CAZyme Identification

**Figure 7:**
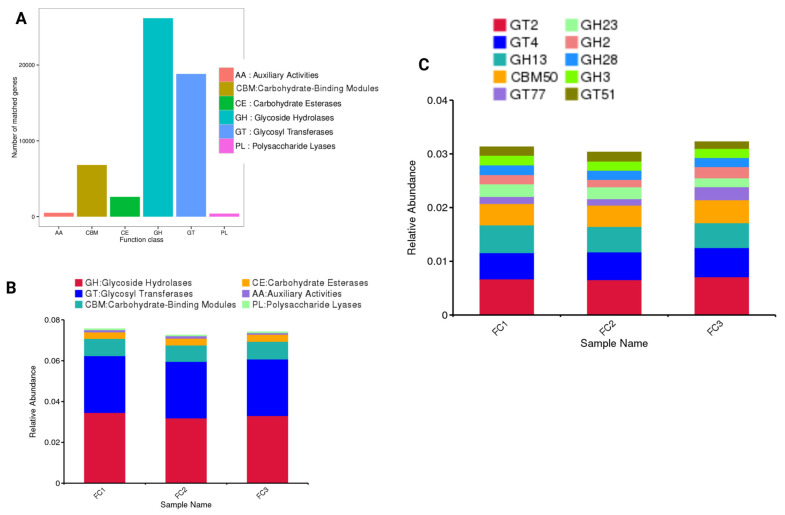
(**A**) The number of annotated genes belonging to each functional class in the CAZy database. (**B**) The relative abundance of the identified CAZyme class and (**C**) the top 10 CAZymes families in the FC1 (Mardan), FC2 (Peshawar), and FC3 (Dera Ismail Khan) samples.

Carbohydrate-active enzymes (CAZymes) play an essential role in the metabolism of carbohydrates and provide a snapshot of the functional potential of the fecal microbiota. Based on the CAZy database analysis, the identified genes were classified into six major functional classes. Overall, glycoside hydrolases (GH) and glycosyl transferases (GT) are the most prevalent, with 26,178 and 18,825 genes, respectively, followed by carbohydrate-binding modules (CBMs). However, more minor counts for carbohydrate esterases (CEs; 2606), auxiliary activities (AAs; 508), and polysaccharide lyases (PLs; 394) were also found (Figure 7A). Similarly, the relative abundance of GH and GT was higher in all sample types, followed by CBMs and other CAZymes (Figure 7B). More specifically, the relative abundance of GT2, GT4, GH13, and CBM50 was higher in all samples compared to other CAZymes (Figure 7C).

## 4. Discussion

It is currently recognized that the GIT microbiota play a central role in maintaining animal health, including disease prevention, by maintaining homeostasis within the GIT associated microbial populations [60]. The GIT microbial community is known to be influenced by various factors, such as diet, age, gender, breed type, and geographical region [19]. While fecal samples do not directly represent these foregut communities, they provide a highly relevant and practical snapshot of the terminal gastrointestinal ecosystem. The fecal microbiota are shaped by upstream digestive processes and reflect the outcome of host–microbe interactions, including the resistant microbial fraction that survives to be excreted. This is particularly crucial for assessing the zoonotic risks and environmental contamination, as feces are the primary source of dissemination. The Khyber Pakhtunkhwa (KP) province in Pakistan encompasses diverse agro-ecological zones, with marked variation in temperature, rainfall patterns, vegetation types, and livestock production systems across districts. These environmental differences are likely to impose distinct selective pressures on the fecal microbiota of cattle. However, region-specific information on the taxonomic and functional profiles of cattle fecal microbiota in KP remains scarce. Therefore, this pilot study employed a metagenomic approach to characterize the fecal microbiota of cattle from three ecologically distinct districts in KP, aiming to identify microbial diversity and functional profile. However, our study provides the first comprehensive taxonomic inventory of the cattle fecal microbiome in three districts in KP, Pakistan. We observed distinct regional patterns, and hence, we acknowledge that the underlying drivers, such as diet, farm management, and climate, remain unexplored in the current study. The higher abundance of taxonomic level(s), for instance, may suggest a dietary influence, but without direct feed composition data, this remains a hypothesis for future testing. Hence, our study should therefore be viewed as a foundational baseline and a hypothesis-generating resource for future studies.

The fecal microbiota of cattle across the three sampling sites was dominated by bacteria, with minor contributions from viruses, archaea, and Eukaryota, a pattern consistent with previous studies on ruminant GIT ecosystems [61,62]. The predominance of bacteria reflects their central role in feed degradation, fermentation, and nutrient cycling in the bovine GIT, while the presence of viral communities, primarily bacteriophages, likely contributes to microbial population regulation and horizontal gene transfer [63]. Archaea, although present at relatively low abundance, are known to play important roles in hydrogen metabolism and methanogenesis, and their detection aligns with their recognized functional significance despite low relative abundance in cattle feces [64]. At the phylum level, the dominance of Pseudomonadota, Bacillota, and Bacteroidota findings were recorded parallel with commonly documented core phyla in cattle fecal microbiota in literature [65]. The higher relative abundance of Pseudomonadota observed in FC1 and FC2 may reflect environmental or management-related influences, as members of this phylum are often associated with diverse metabolic capabilities and adaptability to variable ecological conditions. In contrast, the increased abundance of Bacillota in FC3 may suggest potential differences in dietary inputs, climate, or husbandry practices, as this phylum includes many fiber-degrading and fermentative taxa commonly linked to host energy harvest and GIT homeostasis [19,66]. The presence of minor phyla, such as Verrucomicrobiota and Campylobacterota, further highlights the taxonomic complexity of cattle fecal microbiota, even in low-abundant groups. At the genus level, the dominance of *Acinetobacter*, *Caryophanon*, and *Escherichia*, along with species such as *C. latum* and *E. coli*, indicates a microbial community composed of taxa capable of surviving in fluctuating environmental conditions. The high relative abundance of *E. coli* and other opportunistic pathogens like *P. stuartii* and *Aliarcobacter skirrowii* (a potential zoonotic enteropathogen) in cattle’s feces suggests a GIT environment that may be conducive for the proliferation of commensal or opportunistic pathogens. This could be indicative of subclinical dysbiosis, potentially linked to dietary factors, subacute acidosis, or other stressors. The higher abundance of *Acinetobacter* and *C. latum* in FC1 and FC2 may suggest shared ecological or environmental pressures between these locations, potentially related to climatic conditions, feeding regimes, or microbial exposure from the surrounding environment. The observed regional differences in species richness (e.g., highest in Peshawar) are not just numerical statistics. They may reflect underlying variations in farm management practices, local antibiotic usage, dietary compositions, or environmental exposures. This variation highlights that the fecal microbial ecosystem, and by inference the GIT environment, is plastic and shaped by local conditions, which can have implications for regional disease epidemiology and AMR trends.

In this study, we identified multiple ARGs in all samples belong to different classes, such as *cfxA3*, *bla*_TEM-1B_, *bla*_CTX-M-15_, *bla*_OXA-235_, *bla*_OXA-396_, and *bla*_SHV-187_ of beta-lactam, *aph(3″)-Ib* and *aph(6)-Id* of aminoglycoside, *sul1* and *sul2* of sulfonamide, *dfrA1* and *dfrA14* of trimethoprim, *oqxA*, *oqxB*, *qnrB4*, and *qnrS1* of fluoroquinolones, *catB1* and *catB7* of phenicol class. A similar metagenomics study performed by Ali et al. [35] in other regions of Pakistan also identified multiple ARGs, such as *cfxA3*, *catB8*, *bla*_TEM-1_, *sul4*, *aadA*, and other genes, which are in accordance with the findings of the current study. Another study conducted by Su et al. [66] in China also identified ARGs belonging to tetracycline, aminoglycosides, chloramphenicol, sulfonamides, and others from cattle feces. These findings highlight that cattle feces are an essential reservoir of diverse ARGs belonging to multiple classes and recommend proper treatment before disposal of animal waste to curb the growing threat of AMRs. The *bla*_CTX-M-15_ is a dominant ESBL gene globally, responsible for resistance to cephalosporins, antibiotics that are cornerstone treatments for severe Gram-negative infections. Its presence in our samples, particularly in an agricultural context, underscores the role of non-clinical environments as potential reservoirs and sources of dissemination for clinically relevant resistance that can pose a direct threat to human and animal health. Similarly, the prevalence of *bla*_CTX-M-15_ correlated strongly with the presence of *E. coli* and IncF plasmid replicons. This finding is significant because IncF plasmids are well-characterized vectors for spreading ESBL genes among *Enterobacteriaceae*, a family that includes major human pathogens. This points to a direct pathway for the movement of this resistance from our study samples to clinically relevant bacteria [67]. Furthermore, the identification of *tet*(X) genes, which confer resistance to tigecycline, a last-resort antibiotic, is of critical concern and needs immediate attention. Earlier, such findings have also been reported by Kang et al. [68], who identified a high prevalence of *tet*(X) genes in dairy farm wastewater and cattle feces. The emergence of *tet*(X) outside of clinical settings is a significant public health concern, as it indicates that resistance mechanisms against last-line antibiotics are permeating environmental and animal-associated microbiomes, potentially spreading via the food chain or direct contact. The co-occurrence of high relative abundances of *Acinetobacter* (a genus known to be a primary reservoir for *tet*(X)) with the detection of *tet*(X) suggests that *Acinetobacter* may be the key vehicle, maintaining and potentially disseminating this last-resort *tet*(X) resistance determinant in this ecosystem [69]. Moreover, the identification of *qnrB19*, a fluoroquinolone-resistant gene in the plasmid contig, indicates its potential to spread through horizontal gene transfer. A previous study by Tran et al. [70] reported that *qnrB19* is known to be carried by small plasmids in multiple bacterial species and is known to be transferred by recombination events. This may be the reason for the identification of this gene in the same contig.

The plasmidome analysis revealed that Col and IncF family plasmids were predominantly carried by all samples. However, plasmids belonging to other families, such as IncI, IncY, and IncR, were also detected. A literature review conducted by Shoaib et al. [71] also identified diverse AMR genes and plasmids in bacteria of poultry and other food-producing animals. A similar study with long-read metagenomics conducted by Peng et al. [72] identified high-copy small plasmids in the animal feces and identified some of the ARGs that were plasmid-associated. Therefore, ARGs identified in this study can be plasmid-associated but, due to short-read sequencing, are unable to be detected in the same contigs. Therefore, our study recommends further metagenomics monitoring of ARGs and plasmids using long-read sequencing to explore deeper genomic insights [72,73]. These findings also indicate an active hotspot for horizontal gene transfer (HGT). This finding has profound biological significance: it shows that the cattle GIT, as reflected in feces, serves as a dynamic bioreactor where antibiotic resistance genes can be exchanged and amplified, even in the absence of direct antibiotic selection pressure. This elevates the cattle GIT from a simple digestive organ to a critical reservoir for AMR emergence and dissemination.

Carbohydrate-active enzymes (CAZymes) play an important role in the metabolism of carbohydrates through the gut microbiota [74]. In this study, the relative abundance of GH and GT was found to be high in all samples, followed by CBMs and other CAZymes. More specifically, the relative abundance of GT2, GT4, GH13, and CBM50 was higher in all samples compared to other CAZymes. Similar findings were reported earlier by Ahmed et al. [74], who also identified that GH CAZymes are predominant enzymes in the metagenomics of *Lagopus scotica* GIT microbiota. Moreover, GHs play an important role in the breakdown of complex carbohydrates, including cellulose, hemicellulose, pectin, starch, and oligosaccharides [75]. These types of enzymes help cattle obtain energy from grass, hay, and silage. In contrast, GTs play an important role in the building of complex carbohydrates, such as the building of microbial cell walls, glycoconjugate synthesis, and metabolic salvage. These enzymes are crucial in maintaining a stable and robust microbial community [76]. The high relative abundance of CBMs plays a key role in binding with carbohydrates and prolongs the interaction of its substrate with the catalytic domain for efficient utilization. CBM50, more specifically, binds with peptidoglycan, a component of bacterial cell walls, and chitin, a component of fungal cell walls, which help in bacterial cell wall remodeling and enable the antifungal activity of enzymes, respectively [77]. Furthermore, functional annotations of existing microbial communities for their associated various metabolic pathways should be performed in future studies.

This study gives a clear image of cattle’s GIT microbiota from different geographical regions, which may have a significant influence on microbial evenness. Although the pool-based samples processing in the current study limits the statistical power and generalizing our findings, this should be overcome via biological replicates in future studies. Hence, further comprehensive studies with larger budgets should prioritize the individual-level sequencing to properly characterize the variance within populations and to correlate microbial features with individual host metadata. We acknowledge that by creating district-level composite samples for sequencing, our study design does not permit statistical comparison between districts or inference to the individual animal level. Instead, this approach was used for initial, cost-effective environmental surveillance of the regional microbial and resistome pool. Although shotgun metagenomics enables comprehensive functional pathway reconstruction, the present pilot study was designed primarily to generate a baseline microbial and resistome inventory. Therefore, expanded analyses of metabolic pathways, signal transduction systems, and functional module interactions were beyond the primary scope of this investigation. Future studies incorporating individual-level replication and deeper functional annotation would allow more detailed exploration of metabolic interactions and functional dynamics within the cattle gut microbiome. Additionally, it is recommended to evaluate the microbial diversity of subjects who have a history of proper usage of antibiotic(s) or medication(s). Overall, our analysis of regional composite fecal samples characterizes the collective GIT microbial environment of dairy cattle in these districts. The detection of a substantial and mobile resistome within a predominantly bacterial community indicates that the bovine GIT in this region is not only a site of digestion but also a significant and accessible reservoir for antimicrobial resistance genes. This environmental reservoir sheds continuously and has direct implications for on-farm disease management and the regional epidemiology of AMRs.

## 5. Conclusions

This pilot study provides the first foundational baseline of cattle’s fecal microbiota from three districts, including Mardan, Peshawar, and Dera Ismail Khan in KP province, Pakistan. The results demonstrate high microbial diversity and suggest that geographical and environmental differences may shape fecal microbial composition in cattle. Future research can now correlate these fecal signatures with direct rumen samples, health records, and production data to build predictive models of cattle health and productivity. The prominence of certain opportunistic pathogens in the fecal community warrants investigation into potential sub-clinical GIT health issues affecting the herds. The identification of diverse resistance genes and plasmids in all samples indicated the potential for the spread of resistance genes and plasmids through HGT within and between bacterial microbiota, which is evidenced by the detection of the *qnrB19* gene in the Col440I plasmid contig. The fecal resistome is extensive and mobile, identifying dairy cattle in these regions as important reservoirs for AMR genes capable of spreading via HGT. This pilot survey establishes a baseline of the microbial and resistome profiles in dairy cattle feces from the study regions. Future studies are needed to move from description to mechanistic understanding and application. Priorities may include (1) controlled intervention trials to test dietary strategies for mitigating the observed AMR burden; (2) multi-omics integration to assess the mobility and transcriptional activity of the detected resistance genes; and (3) longitudinal studies correlating specific microbial or resistome signatures with cattle health and production outcomes to validate their diagnostic or prognostic utility.

## Figures and Tables

**Table 1:** The basic information regarding the statistics of scaftigs in the assembly results (≥500 bp) of each sample (FC1: Mardan, FC2: Peshawar, and FC3: Dera Ismail Khan).

Sample ID	Total Length (bp)	No. of Scaftigs	Average Length (bp)	N50 Length (bp)	N90 Length (bp)	Max Length (bp)
FC1	166,648,196	158,307	1052.69	1034	547	150,740
FC2	225,409,088	204,149	1104.14	1090	552	296,846
FC3	301,737,525	318,048	948.72	908	546	506,800

Total length (bp): The total length of the assembled scaftigs. Scaftigs num: The total number of scaftigs assembled. Average length (bp): The average length of scaftigs. N50 length (bp): Sort the scaftigs by length, then add them from long to short, and the length value of the scaftigs when the sum value reaches 50% of the total length of the scaftigs. N90 length (bp): Sort the scaftigs by length, then add them from long to short, and the length value of the scaftigs when the sum value reaches 90% of the total length of the scaftigs. Max length (bp): The length value of the longest scaftigs assembled.

**Table 2:** Resistome and plasmidome acquired by FC1 (Mardan), FC2 (Peshawar), and FC3 (Dera Ismail Khan) samples.

Genomic Features	FC1	FC2	FC3
	Resistome		
β-lactam	*bla*_ACT-16_, *bla*_CTX-M-15_, *bla*_SHV-187_, *bla*_MOX-6_, *bla*_OXA-427_, *bla*_OXA-235_, *bla*_CMH-3_, *bla*_TEM-1B_, *bla*_DHA-1_, *bla*_L1_, *cfxA3*	*bla*_ACT-6_, *bla*_DHA-1_, *bla*_SHV-187_, *bla*_ACI-1_, *bla*_TEM-1B_, *bla*_MIR-1_, *cfx_A3_*, *bla*_OXA-235_, *bla*_CTX-M-15_, *bla*_L1_	*bla*_ACI-1_, *cfxA3*, *bla*_PAO_, *bla*_TEM-1B_, *bla*_CTX-M-15_, *bla*_OXA-235_, *bla*_OXA-396_, *bla*_SHV-187_
Aminoglycosides	*aadA1*, *aph(3″)-Ib*, *aph(6)-Id*, *aac(3)-IIa*, *ant(6)-Ia*, *aph(3′)-IIc*	*aac(2′)-Ia*, *ant(6)-Ia*, *aadA6*, *aph(3″)-Ib*, *aph(6)-Id*, *aph(3′)-IIc*, *aac(3)-IIa*, *ant(6)-Ia*	*aph(3′)-IIb*, *aph(3″)-Ib*, *aph(6)-Id*, *aph(3′)-Ia*,
Sulfonamides	*sul1*, *sul2*	*sul1*, *sul2*, *sul3*	*sul1*, *sul2*, *sul3*
Trimethoprim	*dfrA1*, *dfrA14*	*dfrA1*, *dfrA14*, *dfrG*	*dfrA1*, *dfrA7*, *dfrA14*, *dfrG*
Fluoroquinolones	*oqxA*, *oqxB*, *qnrB4*, *qnrS1*	*oqxA*, *oqxB*, *qnrB4*, *qnrS1*	*oqxA*, *oqxB*, *qnrB4*, *qnrB19*, *qnrS1*
Phenicol	*floR*, *catB1*, *catB7*	*floR*, *catA3*, *catB1*, *catB7*	*catB1*, *catB7*, *cat86*
Tetracycline	*tet(X)*, *tet(40)*, *tet(A)*, *tet(W)*, *tet(L)*, *tet(O)*	*tet(X)*, *tet(39)*, *tet(40)*, *tet(A)*, *tet(B)*, *tet(W)*, *tet(O)*, *tet(L)*	*tet(X)*, *tet(A)*, *tet(B)*, *tet(O)*, *tet(W)*, *tet(32)*, *tet(40)*
Macrolide	*lnu(C)*, *lnu(G)*, *mef(A)*, *msr(D)*	*erm(F)*, *lnu(B)*, *lnu(C)*, *lnu(G)*, *mef(A)*, *mph(A)*	*mef(A)*, *erm(F)*, *lnu(C)*, *msr(D)*
Phosphonic	*fosA*, *fosA6*, *fosA7*	*fosA*, *fosA7*	*fosA*
MDR efflux pump	*mdf(A)*	*mdf(A)*	*mdf(A)*
Plasmidome	IncI1-Alpha, Col3M, Col440I, Col440II, ColRNAI, ColpVC, IncFII(Yp), IncFII, IncFII(pHN7A8), IncFIA, IncFIA(HI1), IncFIB(AP001918), IncFIB(K), IncFIC(FII), IncR, IncY	Col440I, Col440II, ColRNAI, Col(MG828), Col3M, ColE10, ColpVC, IncFII, IncFIA, IncFIA(HI1), IncFIB(pB171), IncFIB(AP001918), IncFIB(K), IncFIC(FII), IncR, IncY	IncI1-Alpha, Col440I, Col440II, ColRNAI, Col(MG828), Col8282, Col156, Col(BS512), Col(IMGS31), Col(IRGK), Col3M, ColE10, ColpVC, IncFII(pSE11), IncFIA, IncFIA(HI1), IncFIB(pB171), IncFIB(AP001918), IncFIB(K), IncR, IncX1, IncY

## Data Availability

The metagenome sequencing data have been deposited in GenBank under BioProject ID: PRJNA1370018.

## Supplementary Materials

The following supporting information can be downloaded at: https://www.mdpi.com/article/10.3390/vetsci13030275/s1; Figure S1: The figure shows the number of genes in each respective sample via a petal plot/Venn diagram. Figure S2: In the figure, the circles represent different taxonomic levels from the inside to the outside (species of each kingdom) in the FC1 sample. The size of the fan represents the relative abundance ratio of the different microorganisms at each taxonomic level. (A): kingdom, (B): phylum, (C): class, (D): order, (E): family, (F): genus, and (G): species. Figure S3: In the figure, the circles represent the different taxonomic levels from the inside to the outside (species of each kingdom) in the FC2 sample. The size of the fan represents the relative abundance ratio of the different microorganisms at each taxonomic level. (A): kingdom, (B): phylum, (C): class, (D): order, (E): family, (F): genus, and (G): species. Figure S4: In the figure, the circles represent different taxonomic levels from the inside to the outside (species of each kingdom) in the FC3 sample. The size of the fan represents the relative abundance ratio of the different microorganisms at each taxonomic level. (A): kingdom, (B): phylum, (C): class, (D): order, (E): family, (F): genus, and (G): species. Figure S5. Observed microbial counts per sample and each taxonomic level. Table S1: Statistical results of data processing. Table S2: Gene catalogue basic information statistics. Table S3: Identity, coverage, and conitgs-level information of each detected AMR gene in all samples.
