# Supplementary material for: Shotgun Metagenomics Reveals Microbial Diversity, Resistome, and Plasmidome in Dairy Cattle Feces
Source: Vet Sci. 2026 Mar 16;13(3):275. doi: 10.3390/vetsci13030275 (PMC13030266; doi:10.3390/vetsci13030275)
Supplement: Supplementary file 1 [file vetsci-13-00275-s001.zip › Supplementary Figures.pdf]

Article

Shotgun Metagenomics Reveals Microbial Diversity, Resistome, and Plasmidome in Dairy Cattle Feces

Shehla Shehla [1], Muhammad Kashif Obaid [2,3], Sadaf Niaz [1], Munir Ahmad Khan [4], Anum Ali Ahmad [5], Mostafa A. Abdel-Maksoud [6], Abdulaziz Alamri [7], Salman Alrokayan [6], Muhammad Shoaib [8,*], Sumaira Shams [1,*] and Qiaoyun Ren [2,3,9,*]

[1] Department of Zoology, Abdul Wali Khan University, Garden Campus Mardan, Mardan 23200, Pakistan; shehla@awkum.edu.pk (S.S.); sadaf@awkum.edu.pk (S.N.)
[2] State Key Laboratory of Animal Disease Control and Prevention, Lanzhou Veterinary Research Institute, College of Veterinary Medicine, Lanzhou University, Chinese Academy of Agricultural Sciences, Lanzhou 730046, China; kashifobaid@awkum.edu.pk
[3] Key Laboratory of Veterinary Parasitology of Gansu Province, Gansu Province Research Center for Basic Disciplines of Pathogen Biology, Lanzhou 730046, China
[4] Department of Medicine, Gomal Medical College, Dera Ismail Khan 29050, Pakistan; munirwensam@gmail.com
[5] The Roslin Institute, The University of Edinburgh, Easter Bush Campus, Edinburgh EH25 9RG, UK; aahmad3@ed.ac.uk
[6] Research Chair of Biomedical Applications of Nanomaterials, Biochemistry Department, College of Science, King Saud University, P.O. Box 2455, Riyadh 11451, Saudi Arabia; mabdmaksoud@ksu.edu.sa (M.A.A.-M.); salrokayan@ksu.edu.sa (S.A.)
[7] Biochemistry Department, College of Science, King Saud University, P.O. Box 2455, Riyadh 11451, Saudi Arabia; abalamri@ksu.edu.sa
[8] Jiangsu Co-Innovation Center for Prevention and Control of Important Animal Infectious Diseases and Zoonoses, College of Veterinary Medicine, Yangzhou University, Yangzhou 225009, China
[9] Hebei Key Laboratory of Animal Physiology, Biochemistry and Molecular Biology, Hebei Collaborative Innovation Center for Eco-Environment, Ministry of Education, Key Laboratory of Molecular and Cellular Biology, College of Life Sciences, Hebei Normal University, Shijiazhuang 050024, China
* Correspondence: 008748@yzu.edu.cn (M.S.); sumairashams@awkum.edu.pk (S.S.); renqiaoyun@caas.cn (Q.R.)

Venn diagram analysis

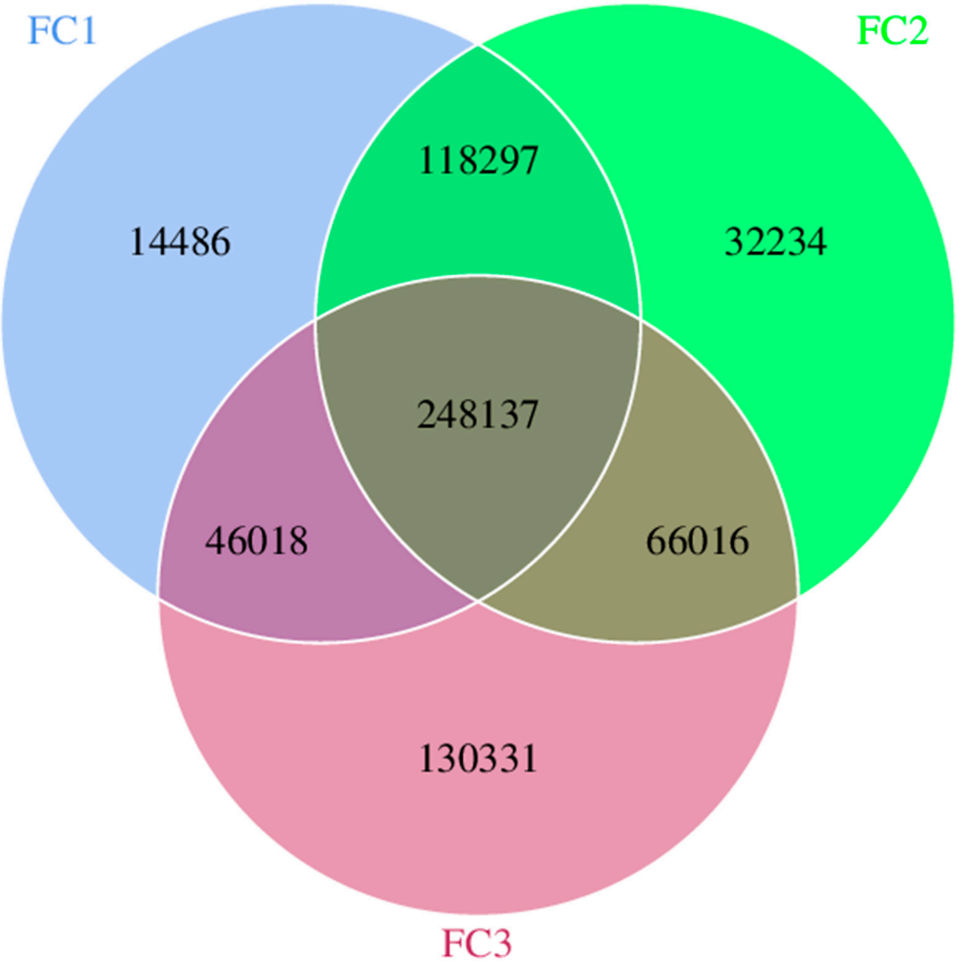

Figure S1. The figure shows the number of genes in each respective sample via petal plot/Venn diagram. (FC1: Mardan, FC2: Peshawar, FC3: Dera Ismail Khan).

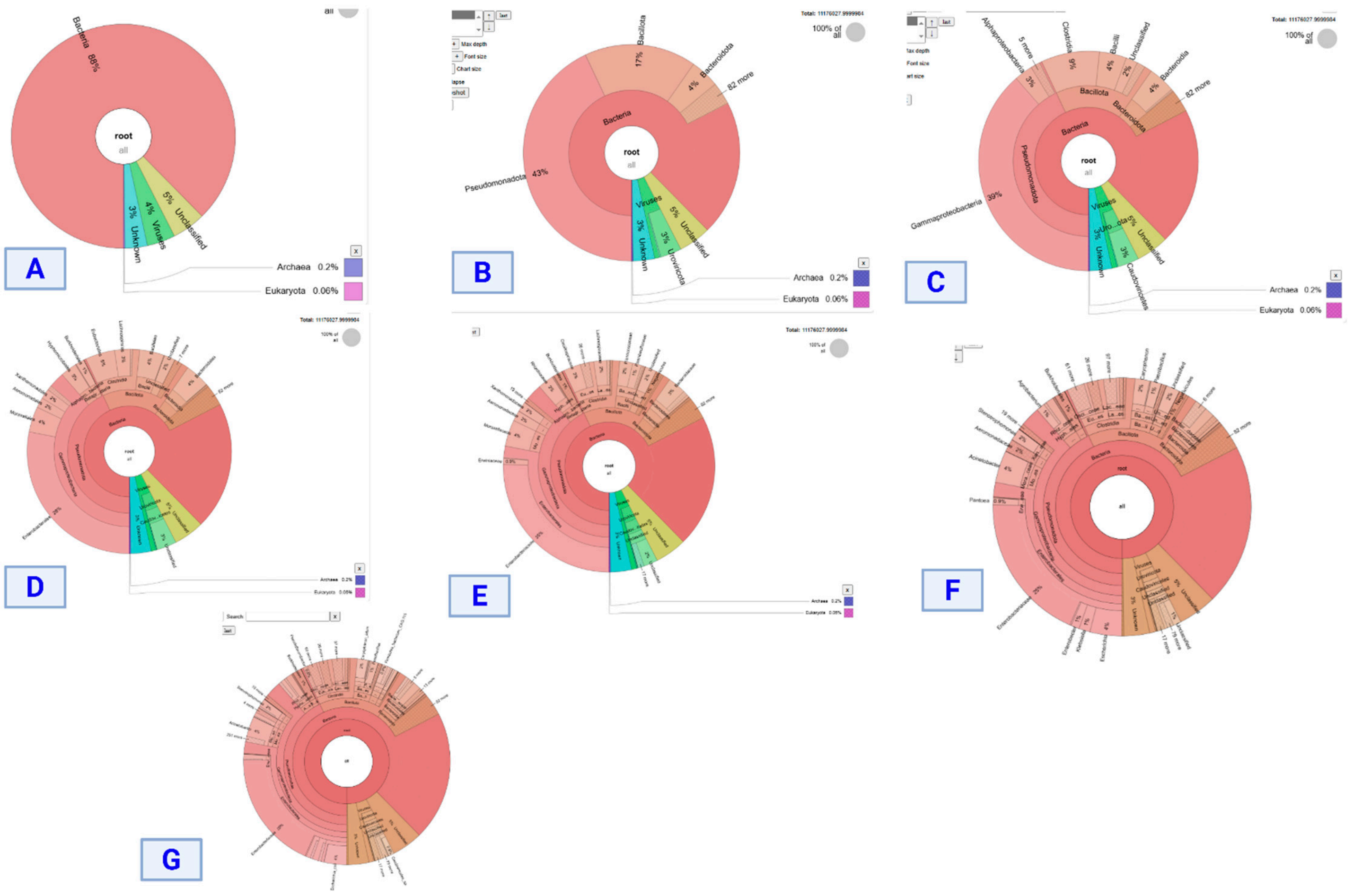

Figure S2. In the figure, the circles represent different taxonomic levels from the inside to the outside (Species of each Kingdom) in FC1 sample (Mardan). The size of the fan represents the relative abundance ratio of different microorganisms at each taxonomic level. (A): kingdom, (B): phylum, (C): class, (D): order, (E): family, (F): genus, and (G): species.

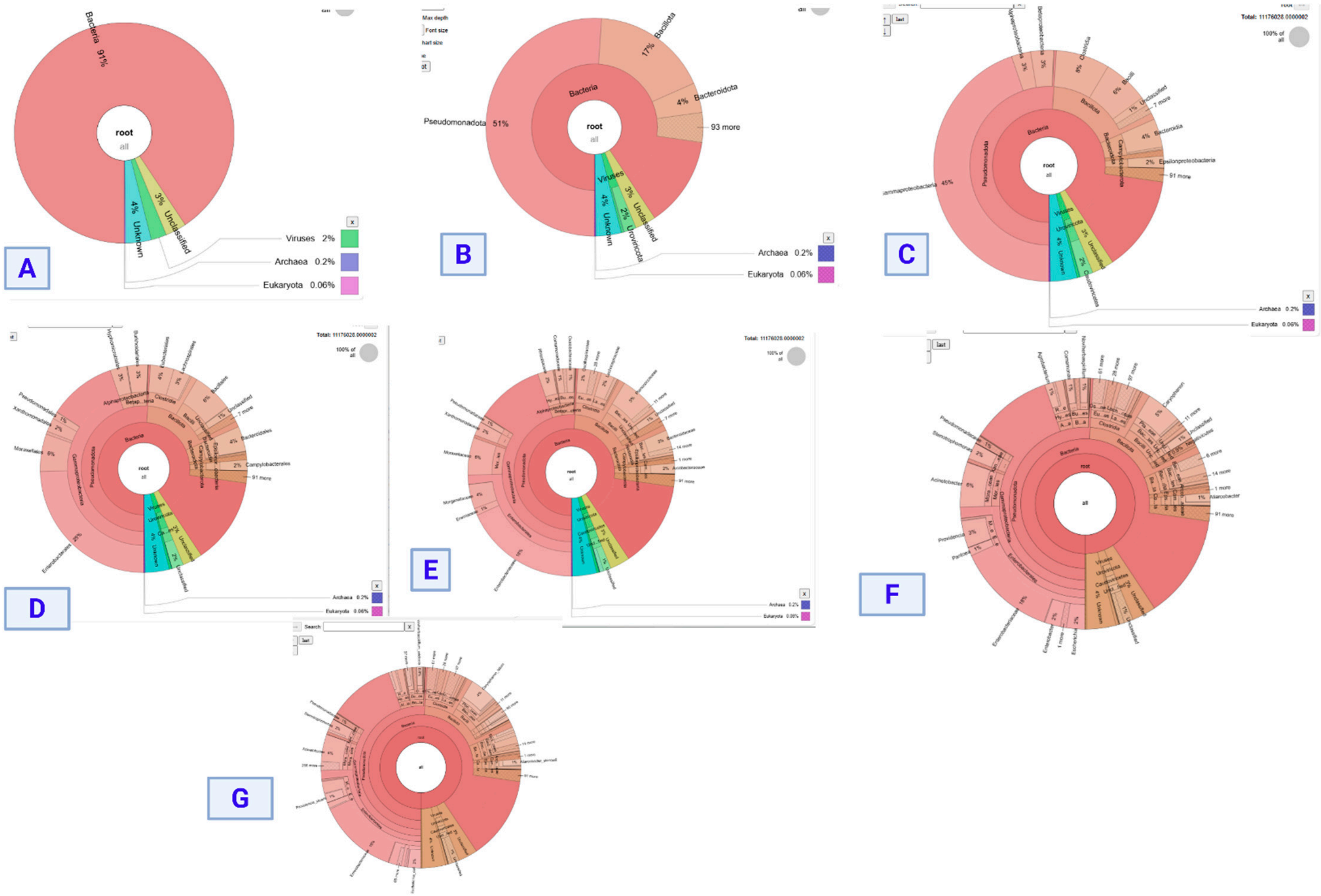

Figure S3. In the figure, the circles represent different taxonomic levels from the inside to the outside (Species of each Kingdom) in FC2 sample (Peshawar). The size of the fan represents the relative abundance ratio of different microorganisms at each taxonomic level. (A): kingdom, (B): phylum, (C): class, (D): order, (E): family, (F): genus, and (G): species.

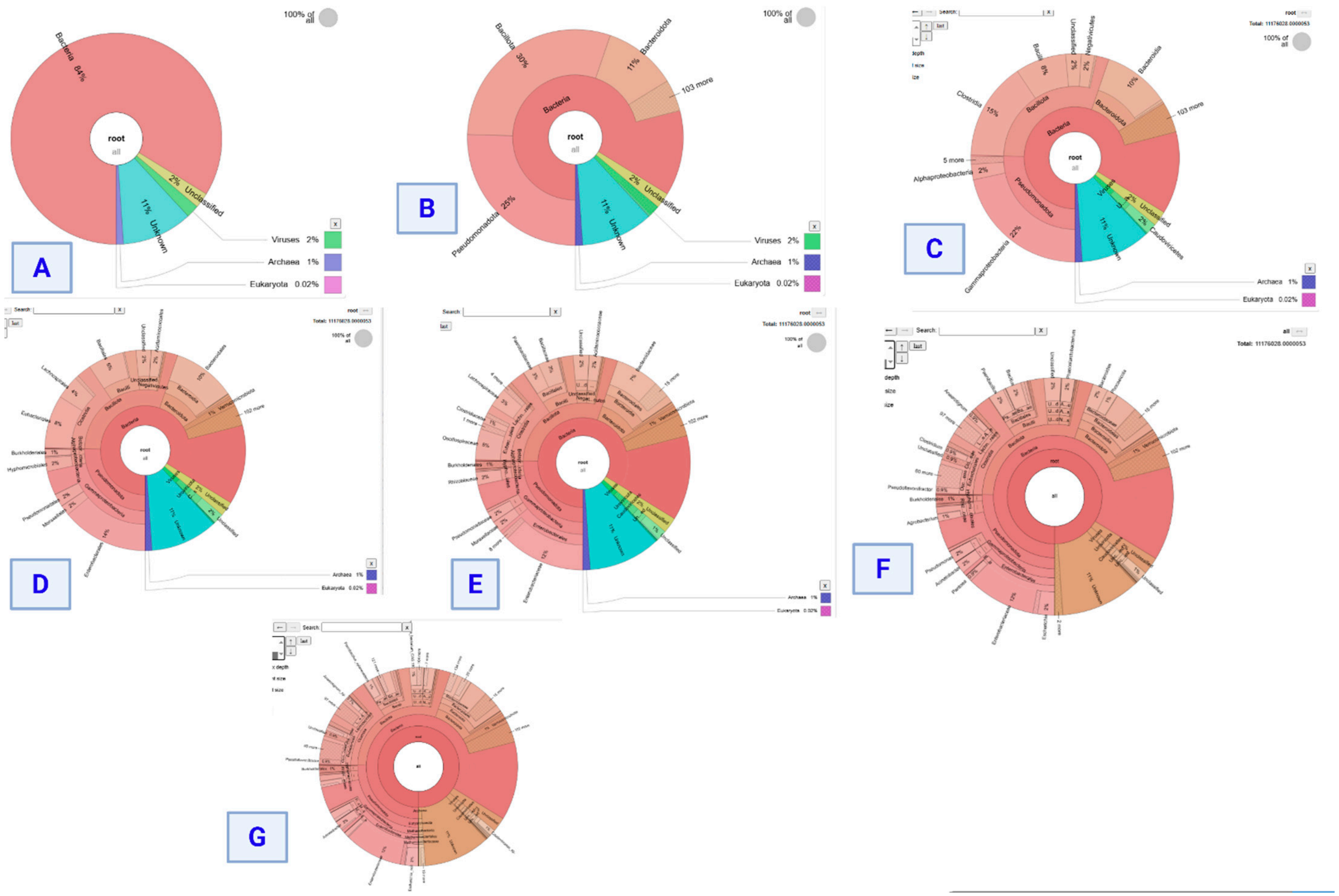

Figure S4. In the figure, the circles represent different taxonomic levels from the inside to the outside (Species of each Kingdom) in FC3 sample (Dera Ismail Khan). The size of the fan represents the relative abundance ratio of different microorganisms at each taxonomic level. (A): kingdom, (B): phylum, (C): class, (D): order, (E): family, (F): genus, and (G): species.

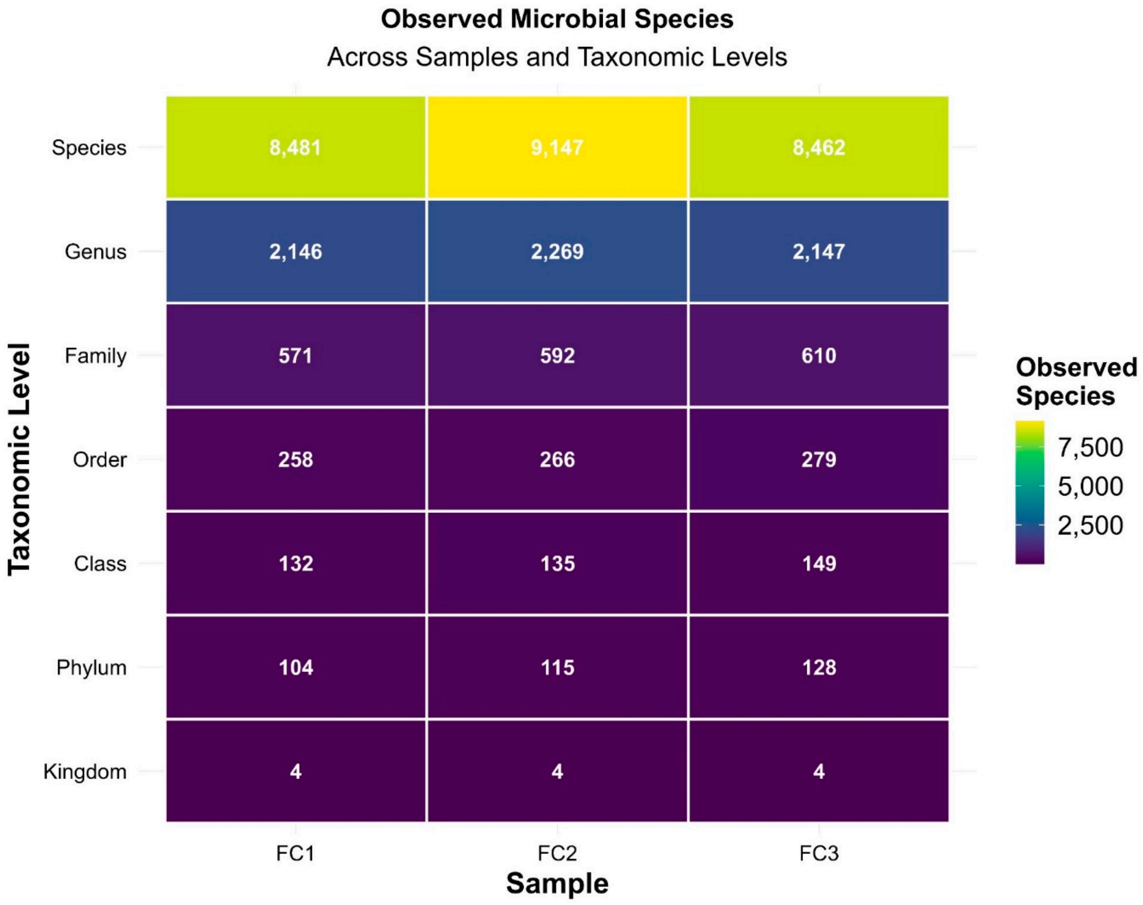

Figure S5. Observed microbial counts per sample and each taxonomic levels. (FC1: Mardan, FC2: Peshawar, FC3: Dera Ismail Khan).
